# Shaping mechanisms of metal specificity in a family of metazoan metallothioneins: evolutionary differentiation of mollusc metallothioneins

**DOI:** 10.1186/1741-7007-9-4

**Published:** 2011-01-21

**Authors:** Òscar Palacios, Ayelen Pagani, Sílvia Pérez-Rafael, Margit Egg, Martina Höckner, Anita Brandstätter, Mercè Capdevila, Sílvia Atrian, Reinhard Dallinger

**Affiliations:** 1 Departamento Química, Faculty Ciències, Universitat Autònoma de Barcelona, E-08193 Cerdanyola del Vallès, Barcelona, Spain; 2 Departamento Genètica, Faculty Biologia, Universitat de Barcelona, Avenida Diagonal 645, E-08028 Barcelona, Spain; 3 Institute of Zoology and Center of Molecular Biosciences Innsbruck (CMBI), University of Innsbruck, Technikerstraße 25, A-6020 Innsbruck, Austria; 4 Division of Genetic Epidemiology, Department of Medical Genetics, Molecular and Clinical Pharmacology, Innsbruck Medical University, Austria

## Abstract

**Background:**

The degree of metal binding specificity in metalloproteins such as metallothioneins (MTs) can be crucial for their functional accuracy. Unlike most other animal species, pulmonate molluscs possess homometallic MT isoforms loaded with Cu^+ ^or Cd^2+^. They have, so far, been obtained as native metal-MT complexes from snail tissues, where they are involved in the metabolism of the metal ion species bound to the respective isoform. However, it has not as yet been discerned if their specific metal occupation is the result of a rigid control of metal availability, or isoform expression programming in the hosting tissues or of structural differences of the respective peptides determining the coordinative options for the different metal ions. In this study, the Roman snail (*Helix pomatia*) Cu-loaded and Cd-loaded isoforms (HpCuMT and HpCdMT) were used as model molecules in order to elucidate the biochemical and evolutionary mechanisms permitting pulmonate MTs to achieve specificity for their cognate metal ion.

**Results:**

HpCuMT and HpCdMT were recombinantly synthesized in the presence of Cd^2+^, Zn^2+ ^or Cu^2+ ^and corresponding metal complexes analysed by electrospray mass spectrometry and circular dichroism (CD) and ultra violet-visible (UV-Vis) spectrophotometry. Both MT isoforms were only able to form unique, homometallic and stable complexes (Cd_6_-HpCdMT and Cu_12_-HpCuMT) with their cognate metal ions. Yeast complementation assays demonstrated that the two isoforms assumed metal-specific functions, in agreement with their binding preferences, in heterologous eukaryotic environments. In the snail organism, the functional metal specificity of HpCdMT and HpCuMT was contributed by metal-specific transcription programming and cell-specific expression. Sequence elucidation and phylogenetic analysis of MT isoforms from a number of snail species revealed that they possess an unspecific and two metal-specific MT isoforms, whose metal specificity was achieved exclusively by evolutionary modulation of non-cysteine amino acid positions.

**Conclusion:**

The Roman snail HpCdMT and HpCuMT isoforms can thus be regarded as prototypes of isoform families that evolved genuine metal-specificity within pulmonate molluscs. Diversification into these isoforms may have been initiated by gene duplication, followed by speciation and selection towards opposite needs for protecting copper-dominated metabolic pathways from nonessential cadmium. The mechanisms enabling these proteins to be metal-specific could also be relevant for other metalloproteins.

## Background

Metallothioneins (MTs) constitute a superfamily of genetically polymorphic cysteine (Cys)-rich polypeptides that bind, with high affinity, closed-shell metal ions such as Zn^2+^, Cd^2+^, Cu^+ ^and others [1,2]. In many organisms they play multiple roles. By serving as principal cellular stores for Zn^2+ ^and Cu^+ ^they warrant the supply of these essential trace elements in growth and rescue processes and, by their high sequestration power, they shield cell components from deleterious bonding by highly reactive metals such as Cd^2+ ^and overabundant Zn^2+ ^and Cu^+ ^[3,4]. In addition, they are also thought to serve as quenchers of free radicals [5,6]. In most animal species examined different MT isoforms show poor or no appreciable differentiation and specialization in their functions and metal-binding preferences [7,8], although variations in metal selectivity between MT domains exist in some cases [9-11]. As a consequence, the metal composition of most native metal-MT complexes is very often remarkably promiscuous [12,13]. Occasionally, apparent metal specificity results from a disproportional oversupply of a certain metal ion, due to particular physiological conditions such as metabolic trace element disorders [14] or cellular overload due to metal exposure [15]. In such cases, this metal ion occupies all the binding sites of an MT molecule which would otherwise form heterometallic complexes. However, true metal specificity requires an exclusive binding preference for a certain metal to an MT peptide due to its innate structural configuration.

Understanding how MTs and other metalloproteins achieved metal specificity through evolution is a key question in the study of their structure/function relationship [2,16]. Among molluscs, pulmonate snails provide an optimal system with which to study the determinants of metal MT specificity in metazoans. Molluscs comprise a range of economically, medically and ecologically significant species and represent one of the most successful animal phyla, having been able to colonize nearly every habitat on earth [17]. Some gastropod molluscs - particularly from the subclass of pulmonate snails - feature MT isoforms that can be isolated from their tissues as homometallic complexes with either Cd^2+ ^or Cu^+ ^[18-21]. Hence, one isoform isolated from Cd-exposed Roman snails (*Helix pomatia*) exhibited an exclusive metal complement of six equivalents of Cd^2+ ^per mol of protein [22], whereas another isoform from the same species contained 12 equivalents of Cu^+ ^[23]. It has been proposed that these two pulmonate MT isoforms serve metal specific tasks related to cadmium detoxification [24-26] or homeostatic copper regulation [27]. Only recently, a third MT isoform, recovered as a mixed Cd^2+^- and Cu^+^-containing complex has been detected in a terrestrial pulmonate but, due to its low abundance, this isoform is probably less important to the snail's metal metabolism [21].

So far, all pulmonate MT isoforms have been obtained as native metal-MT complexes purified from snail tissues, where they are primarily involved in the metabolism of the metal ion species bound to the respective isoform [18,19]. Therefore, it cannot yet be discerned if their specific metal occupation is the result of a rigid control of metal availability, an isoform expression programming in the hosting cells and tissues or of structural differences of the respective peptides determining the coordinative options for the different metal ions. The aim of the present investigation was, therefore, to use the metal-specific snail MTs as model molecules and to test which of the above determinants contributes to the metal specificity in the snail MT system. To this end, the two metal-specific *H. pomatia *(Hp) MT isogenes (*HpCdMT *and *HpCuMT*) were expressed in two different heterologous environments - bacteria and yeast - in order to test their metal binding and functional capacities independent of their native environments. HpCdMT and HpCuMT were recombinantly synthesized in *Escherichia coli *in the presence of zinc, cadmium and copper and the features of the formed metal complexes compared by optical, chiroptical and mass spectrometric analyses; both isoforms were compared for their functional competence in yeast MT-knockout cells. Furthermore, cell- and tissue-specific expression of the two MT isogenes in the snail was scrutinized by *in situ *hybridization techniques and their expression regulation pattern was assessed by real-time polymerase chain reaction (PCR). Finally, the phylogenetic radiation of the gastropod MT family cluster inside the mollusc phylum was assessed and evaluated. Overall, our data provide an unprecedented analysis of the mechanisms determining, at different levels, the specificity of functions of paralogous MTs, suggesting clues to how these could have been achieved through evolution.

## Results

### Analysis of recombinant HpCdMT and HpCuMT metal complexes reveals sequence-determined specialization of metal binding

Recombinant expression of *HpCdMT *and *HpCuMT *in metal-exposed *E. coli *cells was expected to reveal their *in vivo *binding ability for zinc, cadmium and copper, independent of which is the natively coordinated metal ion.

### Binding of Zn^2+ ^and Cd^2+ ^by HpCdMT

**Figure 1:**
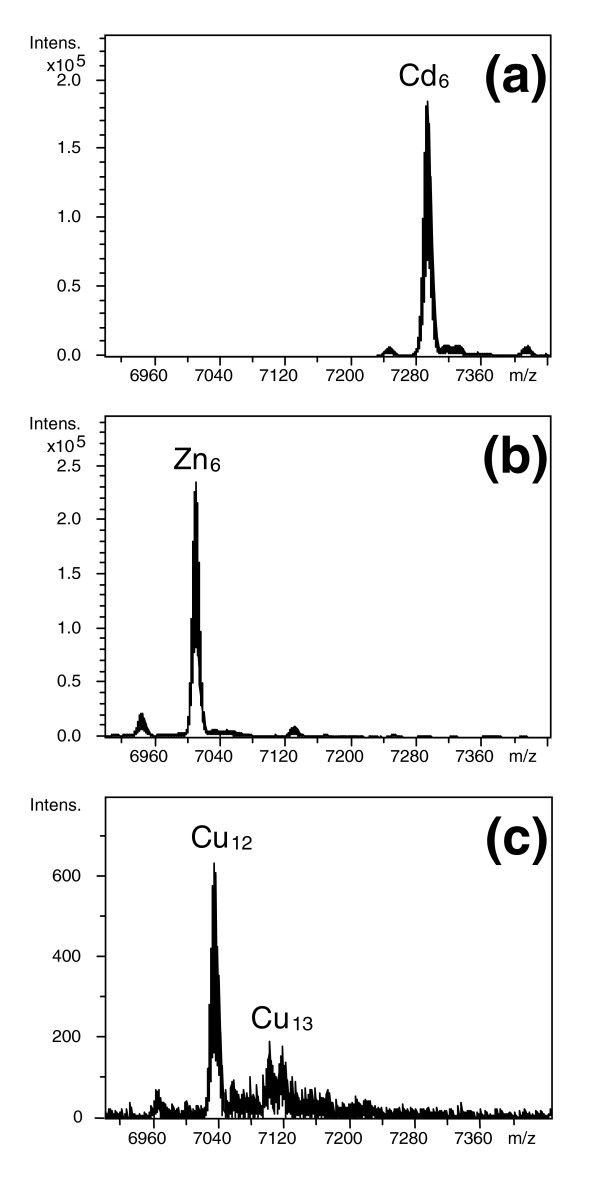
**Deconvoluted electrospray ionization time-of-flight mass spectography spectra of the different metal-metallothionein (MT) complexes recombinantly synthesized**: (a) HpCdMT obtained in Cd-enriched medium; (b) HpCdMT produced in Zn-enriched medium; and (c) HpCuMT synthesized in Cu-enriched medium under low aeration conditions. Inductively coupled plasma atomic emission spectroscopy (ICP-AES) analysis of these preparations indicated a respective mean content of 6.2 Cd/MT in (a) and 5.8 Zn/MT in (b) for HpCdMT and of 12.2 Cu/MT for HpCuMT in (c).

**Figure 2:**
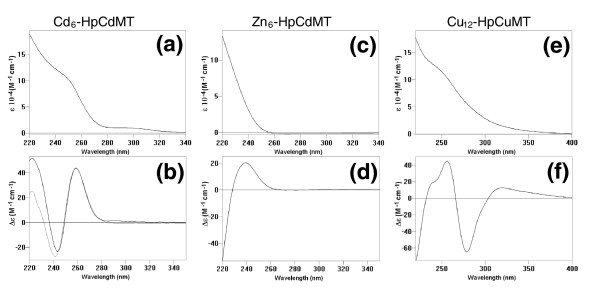
**Ultra violet-*vis *absorption (a, c and e) and circular dichroism (CD (b, d and f) spectra of the following single species recombinantly synthesized in the corresponding metal-enriched media (Cd^2+^, Zn^2+^, and Cu^2+^): Cd_6_-HpCdMT (a, b), Zn_6_-HpCdMT (c, d) and Cu_12_-HpCuMT (e, f).** Panel (b) also encloses (thin line) the CD spectra of the Cd_6_-HpCdMT species prepared *in vitro *by addition of 6 Cd^2+ ^equivalents to Zn_6_-HpCdMT. MT, metallothionein.

Mass spectrometric analysis documents that recombinant synthesis of HpCdMT in *E. coli *cultured in Cd^2+ ^or Zn^2+^-enriched media led to the production of only a single species with a fixed content of either six equivalents of cadmium or zinc (Figure 1a and 1b). The two metal complexes - Cd_6_-HpCdMT and Zn_6_-HpCdMT - display optical spectra with steep rises of absorbance below 270 nm and below 240 nm which is typical of tetrahedral bonding of both metal ions to multiple thiolate ligands (Figure 2a and 2c). The metal-to-sulphur linkages also manifest themselves by the intense positive and negative circular dichroism (CD) bands associated with the absorption envelopes (Figure 2b and 2d). These signals arise in part from the dissymmetric excitonic interactions of the sulphur-based transitions in pairs of doubly coordinated metal-connecting cysteine residues (of bridging thiolate ligands), thereby signifying the collective bonding of Cd^2+ ^or Zn^2+ ^in oligonuclear metal thiolate complexes [28-30]. The spectral features of the recombinant Cd_6_-HpCdMT product are indistinguishable from those of the native Cd_6_-HpCdMT previously isolated from the tissue of Cd-exposed Roman snails [23]. They also reappear when, at neutral pH, the full complement of six equivalents of Cd^2+ ^is added *in vitro *to the metal-free protein apo-HpCdMT [22] or when Zn^2+ ^is replaced in recombinant Zn_6_-HpCdMT by exposure to the much more firmly binding Cd^2+ ^(Figure 2b). Thus, in both *in vivo *and *in vitro*, the structure of the protein product of the *HpCdMT *gene is seen to direct bonding of Cd^2+ ^in a single energetically favoured complex fitting its supposed role of shielding the snail tissue from this highly toxic metal ion [20,24].

### Binding of Cu^+ ^by HpCuMT

The recombinant expression of *HpCuMT *in *E. coli *cultures grown in Cu^2+^-enriched medium led to the formation of homometallic Cu_12_-HpCuMT as an essentially single molecular species (Figure 1c), equivalent to the native complex purified earlier from snail tissue [23]. The absorption spectra display a progressive rise below 350 nm with a broad shoulder centred at 250 nm (Figure 2e) and in CD positive and negative ellipticity bands (Figure 2f). These features match qualitatively and quantitatively those seen in mammalian Cu_12_-MT prepared *in vitro *by adding 12 equivalents of the acetonitrile complex of Cu^+ ^to native MT from rabbit and are attributable to the formation of oligonuclear Cu^+ ^thiolate complexes in trigonal coordination geometry [31]. A molecular species with the same composition and spectral properties was attained *in vitro *by saturating at acidic pH the recombinant, metal-free, apo-HpCuMT peptide with Cu^+ ^using [Cu(CH_3_CN)_4_]ClO_4 _as a titrating agent. As previously observed [23], this homometallic recombinant product is sensitive to atmospheric O_2_. The recombinant complex was formed as a single product only when the bacteria were grown under low aeration conditions [32]. At normal oxygenation the same culture produced a heterometallic mixture of several Cu,Zn-HpCuMT species ranging from M_4 _to M_12_-HpCuMT (where M = Zn + Cu) and with spectroscopic features clearly different from those of Cu_12_-HpCuMT. *In vitro *addition of Cu^+ ^to these species in the form of [Cu(CH_3_CN)_4_]ClO_4 _(see above) also failed to transform these products into the homometallic single form.

### Complexes of HpCdMT and HpCuMT with non-cognate metal partners

**Figure 3:**
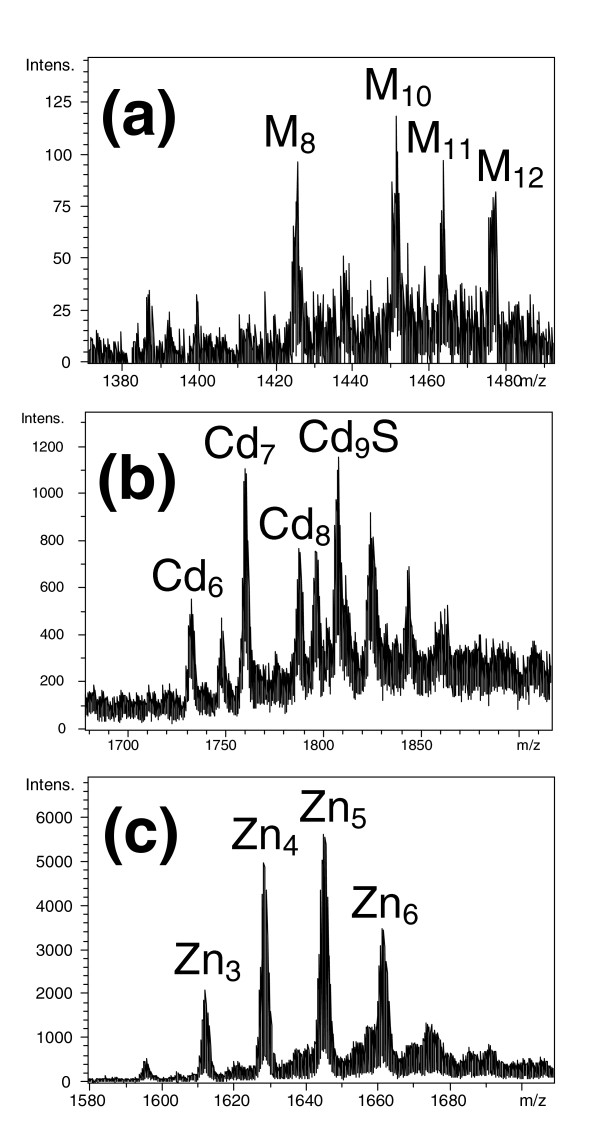
**Electrospray ionization (ESI) time-of-flight mass spectroscopy (MS) spectra of the different metal-metallothionein (MT) complexes obtained when recombinantly synthesizing the MT peptides with their 'non-cognate' metal ion**. (a) HpCdMT produced in Cu-enriched medium and; HpCuMT in (b) Cd- and (c) Zn-enriched media, where M stands for Zn+Cu, due to the difficulty of ESI-MS to discriminate between these two metal ions. For the Zn and Cd-enriched preparations, the MS spectra show the +4 charge state (intensity *vs *m/z) obtained for each preparation, while in the protein produced in a Cu-enriched medium, the MS spectrum shows the +5 charge state (intensity *vs *m/z). inductively coupled plasma atomic emission spectroscopy (ICP-AES) analysis of these preparations indicated a mean content of 0.8 Zn/MT and 7.7 Cu/MT for HpCdMT in (A); 7.8 Cd/MT in (B) and 4.8 Zn/MT in (C) for HpCuMT.

In contrast to the single, well-defined MT species resulting from the recombinant expression of the *HpCdMT *and *HpCuMT *genes in cultures enriched with their cognate metal partners, only poorly defined products were obtained when the partners were interchanged (Figure 3). Thus, expression of the *HpCdMT *gene in the presence of copper resulted in mixtures of heterometallic Zn, Cu-HpCdMT species of varying metal-to-protein stoichiometry, when grown under normal and low aeration conditions. In the first case, their metal complement varied from three to seven equivalents and in the second from eight to 12 equivalents (Figure 3a). The CD spectra of the mixtures (not shown) displayed signals typical of oligonuclear Cu^+^-thiolate bonding but varied in shape and amplitudes in different preparations.

The recombinant synthesis of HpCuMT in Cd^2+^-enriched cultures led to mixtures of a number of Cd-HpCuMT species with a Cd content ranging from six to more than nine equivalents and also including a sulphide-containing Cd_9_S-HpCuMT complex (Figure 3b). The CD spectra of these mixtures (not shown) displayed spectropolarimetric features arising at less than 270 nm from oligonuclear Cd-thiolate complexes and close to 280 nm from sulphide bonding to the Cd-thiolate clusters [33]. In a parallel way, the production of HpCuMT in zinc (Zn)-enriched media yielded mixtures of Zn-containing species ranging from Zn_3 _to Zn_7_-HpCuMT, with the different forms varying in abundance and in different preparations (Figure 3c). The CD spectra were indicative of Zn-thiolate coordination but differed widely in intensity (data not shown).

Therefore, these two MTs behave in accordance with their high metal specificity when recombinantly synthesized by bacteria grown in cultures enriched with the non-cognate metal. The HpCdMT isoform thus rendered several mixed Zn, Cu-MT complexes of different stoichiometries when biosynthesized in a Cu-rich medium. Following an equivalent behaviour, the HpCuMT isoform produced a mixture of species of different stoichiometry when synthesized by bacteria grown on Zn- or Cd-supplemented media. The classification of MTs according to the metal-binding behaviour shown when recombinantly synthesized in cultures enriched with different metals and the correspondence of this classification with other 'metal-specificity' criteria, have been fully reviewed [34].

### Stability of the Cd_6_-HpCdMT and Cu_12_-HpCuMT complexes is documented by their metal exchange inertness

**Figure 4:**
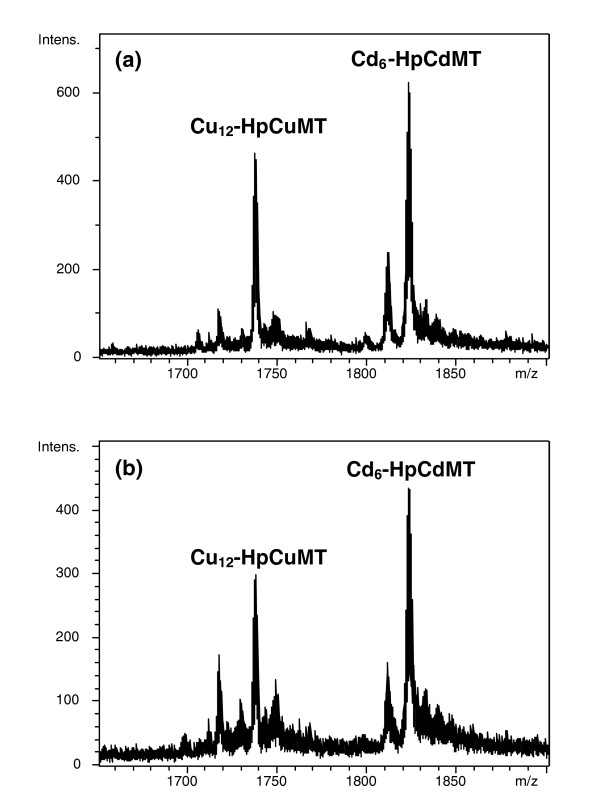
**Electrospray ionization (ESI) time-of-flight mass spectroscopy (MS) spectra of an equimolar mixture of recombinant Cd_6_-HpCdMT and Cu_12_-HpCuMT, recorded at (a) *t *= 0 and (b) *t *= 20 h, showing that no metal exchange occurs between the two HpMT isoforms at 25°C. MT, metallothionein**.

In order to study the lability/inertness of the recombinantly synthesized Cd_6_-HpCdMT and Cu_12_-HpCuMT species and their propensity to exchange their preferentially bound metal ions, an equimolar mixture of these two complexes was allowed to stand for 20 h at 25°C (Figure 4). The invariant electrospray ionization-mass spectrometry (ESI-MS) spectra recorded just after mixing and 20 h later demonstrate that the integrity and individuality of these two species was maintained for a long period of time, which confirms that both metal-HpMT complexes possess an exceptionally high stability and exhibit a persistence attributable to their metal binding specificity.

### Transformation of HpCdMT and HpCuMT in yeast MT-knockout cells confirms metal-specific roles

**Figure 5:**
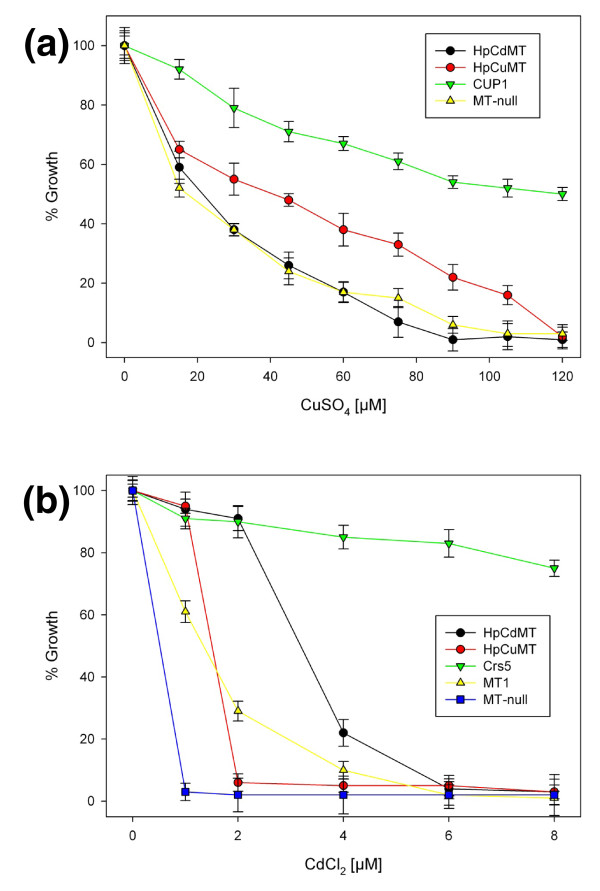
**(a) Copper and (b) cadmium tolerance evaluated by phenotype rescue on DTY4 [metallothionein (MT) deficient] yeast cells**. Metal tolerance of each DTY4 strain transformed with different MTs, as indicated in the side boxes, is shown as a percentage of the growth rate exhibited in a non-metal-supplemented medium. Cup1 and Crs5 are the two yeast MTs, and MT1 stands for the mouse MT1 isoform.

In order to advance from metal-specific folding to metal-specific function, the particular performance of the two snail MT isoforms was compared by complementation studies in another heterologous system. Hence, yeast cells deficient in their endogenous MTs (yeast *Cup1 *and *Crs5 *knockout cells) were transformed with complimentary DNAs (cDNAs) coding for HpCdMT, HpCuMT, mouse MT1, yeast Cup1 and yeast Crs5 and their growth was examined for Cu^2+ ^and Cd^2+ ^tolerance. When these cells were grown at increasing Cu^2+ ^concentrations in the medium (Figure 5a), the highest copper tolerance was observed for the strain transformed with yeast *Cup1*, followed by the strain transformed with the HpCuMT cDNA. The strain transformed with HpCdMT cDNA gave no evidence of tolerating copper at all.

In marked contrast, when the cells were grown in media with increasing concentrations of Cd^2+^, tolerance was greatest in the strain transformed with the endogenous yeast *Crs5 *which reflects the known preference of this yeast MT for divalent metal ions [32]. The second best was the strain transformed with the cDNA for HpCdMT showing a Cd^2+ ^detoxification capacity that was also much better than that of the cells transformed with the cDNA coding for the mouse MT1 isoform, which natively binds either Zn^2+^, Cd^2+ ^or Cu^+^. The strain carrying cDNA for HpCuMT showed almost the same high sensitivity to Cd^2+ ^as the MT-null knockout cells (Figure 5b).

These results show that the two snail MT isoforms also assume metal-specific roles in a heterologous eukaryotic environment (yeast), in accordance with their metal-specific binding preferences revealed by their synthesis in recombinant prokaryotic systems. Significantly, the total equivalence between the features of the metal-MT complexes synthesized in these two hosts (bacteria and yeast) has recently been demonstrated for both cadmium and copper, using the Cup1 MT as a model system [35].

### In pulmonate snails, CdMT and CuMT isoforms are products of cell-specific expression

**Figure 6:**
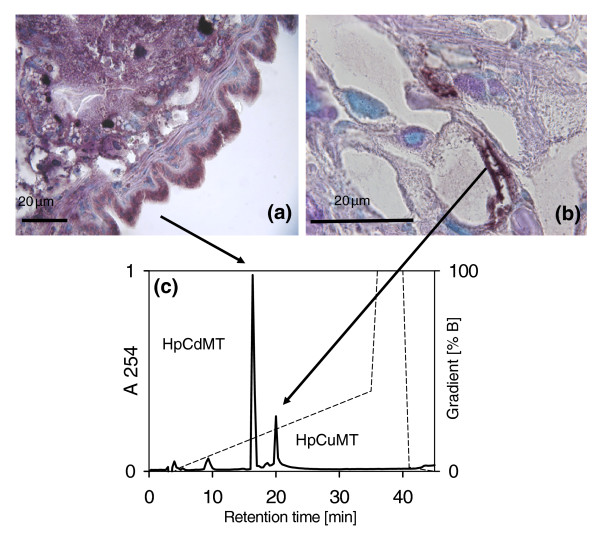
**Cell-specific visualizuation of HpCdMT and and HpCuMT mRNA, and isolation of both native expressed isoforms from snail midgut gland tissue.***In situ* hybridization (dark violet precipitations) of (a) HpCdMT messenger RNA (mRNA) in midgut gland cells and (b) HpCuMT mRNA in rhogocytes from midgut gland of cadmium-exposed *Helix pomatia*. (c) Reverse-phase high-performance liquid chromatogram of purified homogenate supernatants from midgut gland of cadmium-exposed *H. pomatia *snails, showing by the arrows the HpCdMT and HpCuMT isoforms originating from the different cell types, as characterized in references [18] and [19]. MT, metallothionein.

In the midgut gland of the snail *Helix pomatia*, the Cd-specific isoform (HpCdMT) is synthesized in all cell types of this organ (Figure 6a) and is also produced in the epithelial cells of foot, gut and kidney [24]. In contrast, the messenger RNA (mRNA) coding for HpCuMT is located only in one cell type, the so-called rhogocytes (Figure 6b), which are present in the midgut gland, and in many other organs, and have been shown to be the sites of hemocyanin synthesis [27]. Consequently, both metal-specific MT isoforms can be recovered natively from the snail midgut gland (Figure 6c).

### Pulmonate MT isoform genes display metal-specific transcription patterns

**Figure 7:**
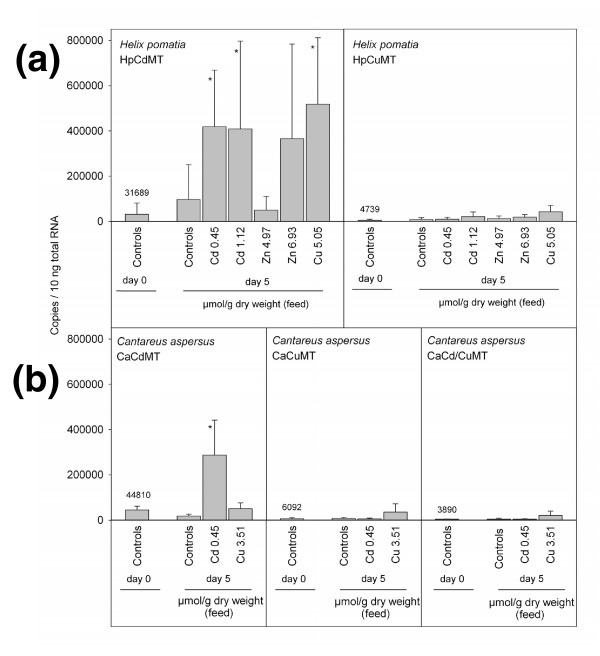
**Real-time detection polymerase chain reaction (copy number/10 ng total RNA) of messenger RNA (mRNA) of *Helix pomatia *HpCdMT and HpCuMT (a) and *Cantareus aspersus *CaCdMT, CaCuMT and CaCd/CuMT (b)**. Respective mRNA concentrations (copy numbers/10 ng total RNA) were measured in midgut gland tissue of control (unexposed) snails at the beginning of the experiments and of controls, as well as metal-exposed snails, after a feeding period of 5 days. For each bar, means and standard deviations are shown (*n *= 5). Asterisks above bars designate significant deviations (*T*-test, *P *≤ 0.05) from control animals at the beginning of the experiment. For controls, copy numbers are specified above bars. Respective metal concentrations in the feed are shown below each bar, expressed as μmol metal/g dry weight of feed. MT, metallothionein.

The pattern of metal-specific transcriptional induction of MT isogenes was examined in two pulmonate species: in *H. pomatia*, the subject of this work, and in *Cantareus aspersus *because, in this species, a third and so far unknown MT isogene (here called *Cd/CuMT*; Figure 7b) has been reported [21]. The product of this gene also seems to occur in other pulmonate snails, as first reported in this work (see below). The effect of metal supplementation in the feed of the snails upon transcription was evaluated by measurement of the mRNA copy number. For both species, the expression of the *CdMT *genes was highly responsive to cadmium exposure (Figure 7a and 7b). While Cd^2+ ^increased the number of transcripts of the *CdMT *genes in both species at concentrations as low as 0.45 μmol Cd^2+^/g dry weight (in the feed), no statistically significant enhancement was observed in *H. pomatia *for a more than a 10-times higher amount of Zn^2+ ^(6.93 μmol Zn^2+^/g dry feed weight; Figure 7a). In Cu^2+ ^a significant increase of the mRNA copies of the HpCdMT gene was seen only at an effective concentration of 5.05 μmol/g dry weight in the feed (Figure 7a). In contrast to the *CdMT *genes, no significant metal-dependent enhancement of mRNA copy number - at least at the metal concentrations assayed - was observed for the *CuMT *and *Cd/CuMT *genes of the two species (Figure 7a and 7b). These induction patterns are totally in accordance with the constitutive expression of the *CuMT *gene in rhogocytes [27], whereas the much higher inducible expression of the *CdMT genes *in epithelial cells supports the view that the product of this gene plays a role in Cd^2+ ^sequestration and detoxification [24-26]. In addition, CdMT may also serve other biological functions, very likely in the form of the Zn^2+ ^complex for *H. pomatia*, as suggested and discussed elsewhere [36].

### HpCdMT and HpCuMT as prototypes of isoform families which have evolved metal specificity in pulmonate snails by modulation of non-cysteine amino acid positions

**Figure 8:**
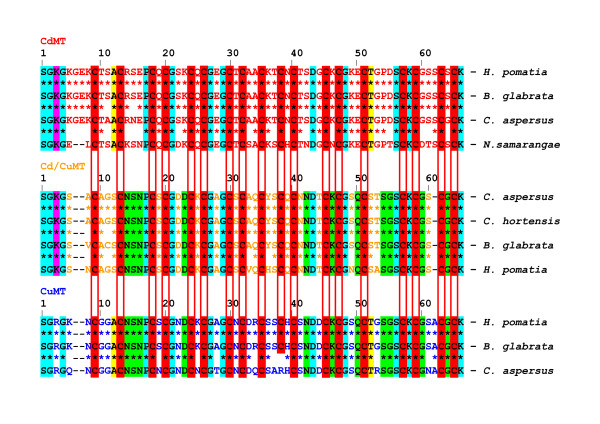
**Amino acid sequence alignments of terrestrial and freshwater pulmonate snail metallothioneins [MTs; CdMT, CuMT and Cd/CuMT]**. Protein or nucleotide sequences were obtained from GenBank and are specified in the legend of Figure 9. Conserved cysteine (cys) positions shared by all isoform families are shaded in red. Conserved non-cys amino acid positions are shaded as follows: blue, shared by all three isoform families; yellow, shared by the CdMT and the CuMT families; pink, shared by the CdMT and Cd/CuMT families; green, shared by the Cd/CuMT and the CuMT families. Asterisks between two sequences indicate identity; Cys residues are marked in bold. For abbreviations of species names see legend of Figure 9.

HpCdMT and HpCuMT can be considered as prototypes of a series of orthologous genes also present, except from *H. pomatia*, in other pulmonate snails. Within molluscs, gastropods and pulmonate snails, in particular, have evolved three MT gene subfamilies, two of them comprising isoforms with a homometallic composition [20,22,23] and distinct metal binding behaviour and functional specificities for either Cd^2+ ^or Cu^+ ^[18 - 21, 23; and this work]. Figure 8 shows an alignment of the, so far identified, MT sequences from pulmonate gastropods, including the secondarily aquatic species *Biomphalaria glabrata*. Throughout, two of the three MT isoforms are alignable with and can thus be assimilated to the known *H. pomatia *isoforms HpCdMT and HpCuMT [18]. The third sequence, the Cd/CuMT isoform first observed in the terrestrial garden snail (*Cantareus aspersus*), has been recovered from native sources as a simultaneously Cu^+ ^and Cd^2+^-containing complex [21] and has been identified in other species too (see Figure 8). However, this isogene is transcribed at low constitutive levels, as it is not inducible by metals at all (Figure 4b) and thus hardly detectable at the protein level. It may, therefore, be only of circumstantial importance for the metal metabolism in its host. However, its discovery is critical for the understanding of the diversification of MTs in this group of organisms.

The three isoform types share strictly conserved Cys positions in their primary structure, confirming the fundamental importance of the sulphur atoms provided by these residues for metal complex and metal thiolate cluster formation, irrespective of the metal species involved. Besides Cys, a few other amino acid positions, either on the N-terminal tail of the peptides or in the direct neighbourhood of Cys residues, show conserved positions through the members of the three isoform subfamilies (Figure 8). In contrast, there is significant variability across the three isoform types for the non-cysteine amino acid residues interspersed between the conserved positions. This suggests that the different metal specificities of the isoforms were achieved by gene duplication and subsequent speciation by evolutionary modulation of these non-coordinating amino acid positions. Moreover, the alignment pattern shows that the similarity between the members of the CuMT and the Cd/CuMT isoform families is clearly higher than that observed between those and the CdMT isoforms (Figure 8, Table 1).

**Table 1:** Comparative protein Blast analysis of pulmonate metallothionein (MT) isoform families*

	Score†	E-value†	Identities	Positives
**CdMT versus CuMT**				
*Helix pomatia*	65.1	8e-17	57%	73%
*Cantareus aspersus*	59.3	5e-15	52%	68%
*Biomphalaria glabrata*	65.1	8e-17	57%	73%

**CdMT versus Cd/CuMT**				
*H. pomatia*	57.8	2e-14	53%	65%
*C. aspersus*	58.2	1e-14	55%	68%
*B.glabrata*	56.2	4e-14	55%	67%

**CuMT versus Cd/CuMT**				
*H. pomatia*	79.3	5e-21	75%	87%
*C. aspersus*	72.0	7e-19	67%	81%
*B. glabrata*	75.1	9e-20	73%	84%

*Protein BLAST calculation (blastp algorithm, NCBI tools) for testing similarities between the three pulmonate MT isoform subfamilies, exemplified by CdMT, CuMT and Cd/CuMT from *H. pomatia*, *C. aspersus *and *B. glabrata*.

**Figure 9:**
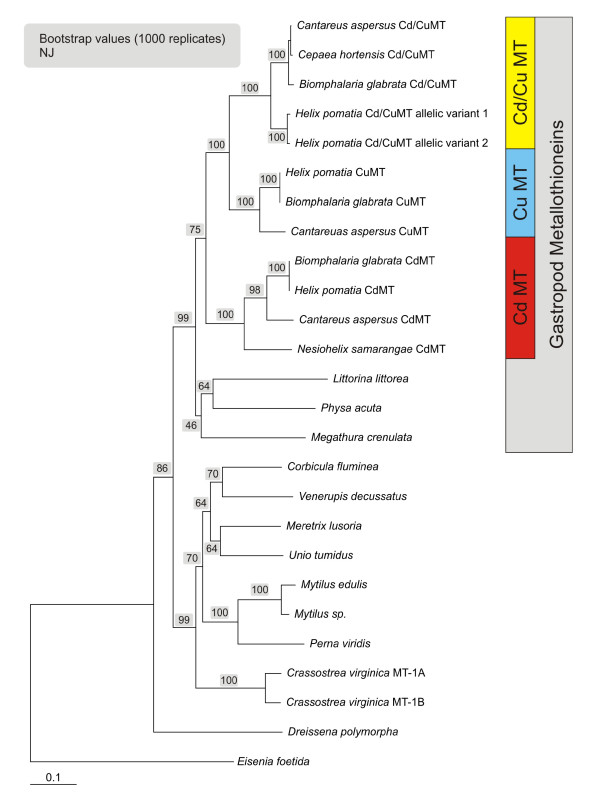
**Nucleotide-based neighbour-joining tree of mollusc metallothioneins (MTs) comprising mussels and gastropods, with *Eisenia foetida *MT used as out-group**. Pulmonate MTs appear grouped in separate clusters of metal-specific subfamilies (CuMTs, Cd/CuMTs and CuMTs). Accession numbers of GenBank entries used were as follows: *Helix pomatia *CdMT (HpCdMT), AAK84863 and AF399740; *H. pomatia *CuMT (HpCuMT), AAK84864 and AF399741. *Biomphalaria glabrata *CdMT, ACS91928 and GQ205374; *B. glabrata *CuMT, ACS91927 and GQ205373; *B. glabrata *Cd/CuMT, ACS91929 and GQ205375; *Cantareus aspersus *CdMT, ABL73910 and EF152281; *C. aspersus *CuMT, ABM55268 and EF178297; *C. aspersus *Cd/CuMT, ABM92276 and EF206312; *Nesiohelix samarangae *CdMT, ACC17831 and EU437399; *Megathura crenulata *MT, AY102647; *Littorina littorea *MT, AY034179; *Mytilus edulis *MT-10, AJ007506 and EF140765; *Crassostrea virginica *MT-1A, AY331697; *C. virginica *MT-1B, AY331699; *Meretrix lusoria *MT, AY525635; *Perna viridis *MT-2, F092972; *Dreissena polymorpha *MT, DPU67347; *Unio tumidus *MT, EF185127; *Corbicula fluminea *MT, EF185126; *Eisenia foetida *MT, AK236886.

**Figure 10:**
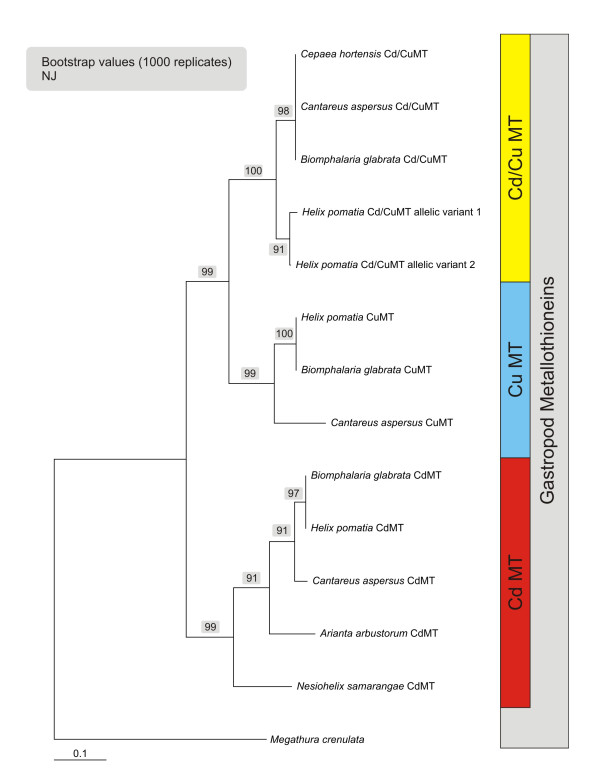
**Amino acid sequence-based neighbour-joining tree of pulmonate metallothionein (MT) subfamilies represented in clusters of Cd/CuMTs, CuMTs and CdMTs, with *Megathura crenulata *MT as out-group **. Accession numbers of GenBank entries are as indicated in the legend of Figure. 9.

A nucleotide-based neighbour-joining tree shows that pulmonate MT isoform subfamilies are assembled in three distinct branches and are thus clearly distinguishable from all other mollusc forms represented by the group of Bivalvia (Figure 9). This suggests that the differentiation into these isoforms has been an evolutionary process which, within molluscs, remained restricted essentially to pulmonate snails. The protein distance analysis tree (Figure 10) confirms the close relationship between the CuMT and Cd/CuMT isoforms (Table 1), which apparently evolved from a common ancestor that gave rise to the differentiation of the Cu-specific and the less metal-specific Cd/CuMT gene subfamilies, clearly segregated from the CdMT gene subfamily. On the other hand, the three pulmonate MT subfamilies share a common root with all other gastropod MTs (Figures 9 and 10).

## Discussion

In MTs, metal binding and metal exchange reactions are mainly governed by the coordination chemistry of thiolate bonding with closed-shell metal ions such as Zn^2+^, Cd^2+^, Cu^+ ^[12]. To be more precise, the relative order of *in vitro *metal binding affinities of apo-MT peptides, as well as the order of displacement capacity of each heavy metal ion within a metal-MT complex (Hg(II) > Cu(I) ~ Ag(I) > Cd(II) > Pb(II) > Co(II) > Zn(II)), follow the rules established for metal-thiolate and metal-sulphide low-molecular mass complexes [37]. However, the assumption that these 'inorganic chemistry' rules are the unique responsible of the metal-MT complex properties would lead to the conclusion that MT polypeptides sharing the same number and position of Cys residues would exhibit equivalent metal binding behaviour. However, this is essentially untrue, as firmly demonstrated in this work for the snail MT system. MT metal specificity is a subject of vivid, current debate [16], as to a larger or lesser extent, all MTs show a degree of metal specificity in native and/or *in vivo *environments. In this case, metal specificity is understood to be the set of determinants that eventually leads a given MT peptide to natively discriminate among metal ions, thus allowing the formation of particular metal complexes and the performance of the biological function for which they were selected. To date, major factors claimed to explain metal-MT specificity in live systems are protein sequence optimization, metal ion availability and/or metal-responsive transcription programming [2].

The structural features of the resulting MT complexes with different metal ion species arise from the equilibrium between kinetic and thermodynamic requirements [29], so that they converge to a stoichiometric ratio that reflects their energetically most stable state [38]. Hence, the observation by ESI-MS of metal-MT species synthesized in *in-vivo *environments, either native or recombinant, allows one to read the propensity of the respective peptides to form metal complexes that are uniquely defined from a stoichiometric and thermodynamic point of view [2,23,34]. In combination with spectroscopic studies, this leads to a clear appraisal of the distinctness of a MT metal specificity, as exemplified in the present work. It can, therefore, be concluded that the homometallic and unique composition of the complexes formed by HpCdMT and HpCuMT with their cognate metal species upon isolation from recombinant cultures (Figure 1) reflects the innate metal specificity of the two isoforms, rather than being the result of an occasional association with metal ions governed by their intracellular or environmental availability. This is confirmed by the poor metal-binding behaviour of the same isoforms confronted with their non-cognate metal ions (Figure 3) and by the inertness of the two complexes, Cd_6_-HpCdMT and Cu_12_-HpCuMT, to exchange metal ions (Figure 4). Such metal ion exchange processes have repeatedly been reported in MTs [39] and would be reasonable to expect [12].

The complete sequential identity of Cys residues and the high degree of conserved positions for other amino acids shared among the three isoform families (Figure 8) suggest, along with their nearly equal size, that metal-specific differentiation of pulmonate snail MTs must have been initiated by gene duplication events, followed by modulatory speciation of amino acid residues located between the cysteine positions. Gene duplication seems to be a common mechanism driving the evolutionary differentiation of MT isoform in animals and is documented for MTs of *Drosophila melanogaster*, among others [40], and the mussel *Crassostrea gigas *[41]. Once duplicated, such genes are free to independently generate mutations, upon which selective forces can then act towards evolution of specific features [42]. The example of pulmonate snail MTs also proves that the evolutionary variation of non-cysteine residues can impose a metal-specific character on to the coordination chemistry of a MT peptide. At present, it is not known how this is achieved at a structural level. However, it must be supposed that, due to their particular position in the sequence and chemical nature of their side-chains, non-cysteine amino acids constrain the sulphur ligands provided by the conserved Cys positions to assume only one of several theoretically possible spatial coordination foldings. Determination of the three dimensional structure of the Cd_6_-HpCdMT and Cu_12_-HpCuMT complexes is actually in progress which may unveil the detailed structural basis of the metal specificity of the two HpMT isoforms.

Cell-specific expression may also contribute to enhancing distinct metal-related functionality [2]. Roman snails, for example, synthesize HpCuMT exclusively in rhogocytes (Figure 6b), the modified cells of mesodermal origin found in virtually all connective tissues of mollusc organs [43]. Since they are also the sites of hemocyanin synthesis [44], it was suggested that HpCuMT functions as a Cu^+ ^reservoir/donor for the nascent hemocyanin [27]. The constitutive expression of *HpCuMT *(Figure 7) and the exclusive preference of this isoform for Cu^+ ^(Figure 6c) support this presumed function. The supposed incorporation of Cu^+ ^into the structure of hemocyanin must occur under reducing conditions, which is also consistent with the high susceptibility of native Cu_12_-HpCuMT complexes to oxidization [20,23] and the fact that homometallic Cu_12_-HpCuMT synthesis is only achieved in low-aerated recombinant cultures (this work). The apparent connection between the tasks of Cu-specific MT isoforms and their presence in organisms with Cu-depending hemocyanins is reminiscent of the situation reported for decapod crustaceans [45,46]. In these animals, concentrations of Cu-MT complexes fluctuate with the metabolic state and the hemocyanin levels during the moulting cycle [47,48]. Cu-specific MTs are also observed in organisms of other kingdoms, especially in fungi [49-51], where their role may be connected to the synthesis of the Cu-containing enzyme tyrosinase, as in *Neurospora crassa *[52].

After exposure of Roman snails to Cd^2+^, virtually all of this metal in the digestive tissues was bound to HpCdMT (20) in a similar manner as for CdMT isoforms of other snail species [53]. Consistently, in the Roman snail, HpCdMT is produced in digestive and excretory tissues [24] (Figure 6a), where the corresponding gene is selectively upregulated by Cd^2+ ^exposure (Figure 7). This suggests that absorption of toxic Cd^2+ ^from the surrounding substrate via the alimentary tract may constitute a particular physiological challenge, exacerbated by the evolutionary transition of gastropods to terrestrial life [36]. Moreover, the sensitivity to Cd^2+ ^of important Cu-dominated metabolic pathways [54-56] and Zn-dependent enzymes [57] could have been the basis of the generation of a specific MT isoform devoted to Cd^2+ ^detoxification in these animals. Our data also demonstrate the ready formation of homometallic complexes of HpCdMT with Zn^2+^, which may be a consequence of the comparable coordination preferences of these two d^10 ^metal ions. The much weaker bonding of Zn^2+ ^[58] to this isoform, however, does not prevent the peptide from functioning as a most effective Cd^2+ ^sequestration agent. In the presence of Zn^2+ ^and the absence of Cd^2+^, the HpCdMT isoform is expressed only at low basal concentrations (Figure 7) and, as in the case of mammalian MTs [59], is thought to serve other functions [36].

## Conclusion

Overall, the present study, together with the extensive body of evidence provided by our previous work, suggests that the pair of the metal-specific *H. pomatia *MT isoforms (HpCdMT and HpCuMT) can be regarded as the prototype of a series of paralogous forms present in pulmonate gastropods. In these organisms, divergent evolution by gene duplication, with subsequent modulation of non-cysteine amino acid positions and a cell specific occurrence and gene expression regulation, has led to the complete separation of their metal-binding preference, cell-specific occurrence, expression regulation and functionality. This resulted in genuine CdMTs becoming inducible forms specializing in the global protection of the organism from the non-essential toxic element Cd and in genuine CuMTs becoming constitutive forms supplying the essential element Cu. Our findings provide experimental evidence and possible answers to how metallotproteins in general, and MTs specifically, were able to achieve partial or complete specificity in their metal binding behaviour and functionality.

## Methods

### Animals and metal exposure

Roman snails (*H. pomatia *L.) were obtained from a commercial dealer (Exoterra, Dillingen, Germany). Garden snails (*C. aspersus*) were provided by the Department of Chrono-Environment, University of Franche-Comté, Besançon, France. All animals were reared under laboratory conditions (20°C, 80% humidity, 12:12 h photoperiod) at the Institute of Zoology in Innsbruck, Austria. Twenty-five snails from each species were split equally into five groups and fed over a period of 5 days on metal-enriched lettuce (*Lactuca sativa*). Metal loading of feed was achieved by soaking salad leaves in a corresponding metal salt solution (CdCl_2 _in H_2_O, with 1 and 3 mg Cd^2+ ^L^-1^; ZnCl_2 _in H_2_O, with 5 and 10 mg Zn^2+ ^L^-1^; CuCl_2_, with 10 mg Cu^2+ ^L^-1^) [60]. Resulting metal ion concentrations in the salad feed were as follows (means ± standard deviation, *n *= 5): Cd^2+^, 0.45 ± 0.11 and 1.12 ± 0.23 μmol g^-1 ^dry weight; Cu^2+^, 3.51 ± 0.73 or 5.05 ± 0.97 μmol g^-1 ^dry weight; Zn^2+^, 4.97 ± 3.42 and 6.93 ± 0.89 μmol g^-1 ^dry weight). These concentrations range from physiologically to moderately elevated levels and are, therefore, representative for what could be the natural conditions encountered by snails. At days 0 and 5, RNA was extracted from the small midgut gland tissue aliquots (~10 mg fresh weight) of at least three animals and processed for cDNA synthesis as detailed below.

For *in-situ*-hybridization of HpCdMT isoform mRNAs, five individuals of *H. pomatia *were exposed over 14 days to a concentration of 14.97 μmol Cd g^-1 ^dry weight. At the end of the exposure period, animals were sacrificed and their organs (midgut gland, midgut, kidney, mantle and foot) used for *in-situ*-hybridization analysis as described below.

### Metal analyses

Metal-enriched salad samples were oven-dried at 60°C for several days. Dried samples (snail tissues: 50-100 mg dry weight; salad samples: 100 - 500 mg dry weight) were wet-digested in screw-capped polypropylene tubes (Greiner, Kremsmünster, Austria) with a mixture of HNO_3 _(suprapure; Merck, Darmstadt, Germany) and distilled water (1:1) by heating at 70°C for several days. At the end of digestion, a few drops of H_2_O_2 _were added to the heated samples. The remaining solutions were diluted to a known volume with distilled water and analysed for metal concentrations (Cd, Zn, Cu) either by flame (model 2380 instrument, Perkin Elmer, Massachusetts, USA) or graphite furnace atomic absorption spectrophotometry (Hitachi Z-8200) with polarized Zeeman background correction (Hitachi, Tokyo, Japan).

### Real-time detection PCR

RNA sampling for real-time detection PCR was done in control snails and animals exposed to metals over a 5 day period (see above). This time range was chosen because, in pulmonate snails, maximal induction of the *CdMT *gene by Cd^2+ ^is reached only after several days. Total RNA was isolated from the homogenized midgut gland tissue of *H. pomatia *and *C. aspersus *individuals (Ultra Turrax T25, IKA Maschinenbau, Staufen, Germany) and quantified after DNaseI digestion (Fermentas, St Leon-Rot, Germany) by means of RiboGreen^® ^RNA Quantitation Kit (Molecular Probes, OR, USA) with calibration curves derived from RNA standards using a fluorescence plate reader (Molecular Devices, CA, USA). Of total RNA, 450 ng was applied for cDNA synthesis using *RevertAid™ H Minus M-MuLV Reverse Transcriptase *(Fermentas) with hexamer primers in a 50 μL approach. Quantification of **the **RNA copy number was performed on a 7500 real-time PCR (RT-PCR) instrument from Applied Biosystems (CA, USA) using the Power SYBR Green approach (Applied Biosystems). Calibration curves from amplicon plasmids were used for copy number analysis for each of the MT isoforms involved, using primers designed with the Primer Express 3.0 software (Applied Biosystems) based on the known cDNA sequences for MT isoforms published in GenBank (*H. pomatia *HpCdMT and HpCuMT, accession numbers: AF399740 and AF399741, respectively; *C. aspersus *CaCdMT, CaCuMT and CaCd/CuMT, accession numbers: EF152281, EF178297, and EF206312). PCR primers used were as follows: HpCdMT: sense primer, 5'AAAGTGCACCTCAGCTTGCA 3'; antisense primer: 5' GCAGGCGGCACA TGTACAG 3'; amplicon length, 85 bp. HpCuMT: sense primer, 5' CCTTGCAGCTGTGGT AACGA 3'; antisense primer, 5' CAAGAACTGCATCGGTCACAA 3'; amplicon length, 65 bp; CaCdMT: sense primer, 5' GCCGCCTGTAAGACTTGCA 3'; antisense primer: 5' CACG CCTTGCCACACTTG 3'; amplicon length, 56 bp. CaCuMT: sense primer, 5' AACAGCAACCCTTGCAACTGT 3'; antisense primer, 5' CGAGCACTGCATTGATCACAA 3'; amplicon length, 74 bp. CaCd/CuMT: sense primer, 5' TGTGGAGCCGGCTGTTCT 3'; antisense primer, 5' CAGGTGTCATTGTTGCATTGG 3'; amplicon length, 59 bp. Optimal primer concentrations were determined by means of dissociation curves established for each primer pair. Two microlitres of cDNA were applied for RT detection PCR in a 20-μL approach (1x Power SYBR Green PCR Mastermix, 1x U-BSA, 900 mM sense primer, 300 mM antisense primer for HpCdMT and HpCuMT; 300 mM sense and 900 mM antisense primer for CaCdMT; 900 mM for sense and antisense primer for CaCuMT; 99 mM sense and 300 mM antisense primer for CaCd/CuMT). The PCR conditions were as follows: 50°C, 2 min; 95°C, 10 min; 40 repeats of 95°C, 15 s; and 60°C, 1 min.

### *In situ *hybridization techniques

Cell- and tissue-specific expression of both HpMT isoforms was demonstrated by *in situ *hybridization (ISH). Construction of digoxigenin-11-UTP-labelled sense and antisense RNA probes for ISH of both MT isoform mRNAs, as well as ISH, antibody exposure and staining of parafomaldehyde-phosphate buffered saline (PBS)-fixed paraffin sections (5 μm) from tissues (midgut gland, midgut, kidney, mantle and foot) of control and metal-exposed animals were performed exactly as described previously [24]. Control sections (exposed to either hybridization antisense or sense probes) were treated and incubated in the same way as samples but without anti-digoxigenin-alkaline phosphatase antibodies. For microscopy, all sections were embedded in Entellan (Merck) [24].

### Construction of the recombinant expression vectors for wild-type Roman snail MT isoforms

The *H. pomatia *coding regions for both MT isoforms were amplified by PCR using the respective cDNAs synthesized during a previous investigation as a template [24]. In order to facilitate their in frame cloning into the pGEX-4T1 expression vector (Amersham GE Healthcare Bio-Sciences AB, Uppsala, Sweden), *Bam*HI and *Sal*I restriction sites were generated just before the anti-thymocyte globulin (ATG) and after the stop codon. The oligonucleotides used for these PCR amplifications were: 5' ACAGGATCCGGACGAGGAAAGAACTGC 3' and 5' ATTGGATCCGGGAAAG GAAAAGGAGAAAAGTG 3' as HpCuMT and HpCdMT upstream primers, and 5' AGGCGTCGACTTGTCGTTTATTTGCAG 3' and 5' ATGCGTCGACTTGTCCTGC GGTTACT 3' as the HpCuMT and HpCdMT downstream primers. 35-cycle PCR reactions were performed under the following conditions: 94°C 30 s, 55°C 30 s and 72°C 30 s, using Deep Vent (New England Biolabs, Massachusetts, USA) thermostable DNA polymerase. PCR products were isolated from 2% agarose gels, digested with *Bam*HI-*Sal*I restriction enzymes (New England Biolabs) and cloned into the corresponding sites of pGEX-4T-1, for glutathione-S-transferase (GST)-MT fusion protein synthesis. The product resulting after purification was used in the second PCR as reverse megaprimer together with the forward primer mentioned earlier. In the final amplification product, the desired mutation had been introduced and the flanking restriction sites (*Bam*HI and *Sal*I) allowed the cloning in frame in pGEX-4T-1. Prior to the protein synthesis assays, all the DNA constructs were confirmed by automatic DNA sequencing (ABI 370, Perkin Elmer Life Sciences), using BigDye Terminator (Applied Biosystems). DH5α was the *E. coli *host strain used for cloning and sequencing purposes and, thereafter, the expression plasmids were transformed into the *E. coli *protease-deficient strain BL21 for recombinant protein overexpression.

### Recombinant synthesis and purification of the metal-HpMT complexes

All HpMT metal complexes analysed in this work were biosynthesized in 2-L Erlenmeyer cultures of the corresponding transformed *E. coli *cells grown in LB medium with 100 mg mL^-1 ^ampicillin and the following metal supplements: 300 μM ZnCl_2 _or CdCl_2 _for the zinc- or cadmium-rich media, or 500 μM CuSO_4 _for the copper-rich medium. Copper cultures were performed under two aeration conditions (high and low aeration) as previously described [32]. GST-MT synthesis was induced with isopropyl-1-thio-β-D-galactopyranoside at a final concentration of 100 mM 30 min before the addition of the metal solution. After a 2.5 h-induction, cells were harvested by centrifugation. In order to prevent oxidation of the metal-HpMT complexes, argon was bubbled in all the steps of the purification following cell disruption.

For protein purification, cells were re-suspended in ice-cold PBS (1.4 M NaCl, 27 mM KCl, 101 mM Na_2_HPO_4_, 18 mM KH_2_PO_4_)-0.5% v/v β-mercaptoethanol, disrupted by sonication and centrifuged at 12,000 *g *for 30 min. The recovered supernatant was used to purify the GST-HpMT polypeptides by batch affinity chromatography with glutathione sepharose 4B (GE Healthcare, Buckinghamshire, UK) incubating the mixture with gentle agitation for 60 min at room temperature. After three washes in PBS and, since the GST-HpMT fusions include a thrombin recognition site, this protease was added (10 μ per mg of fusion protein) and digestion was carried out overnight at 23°-25°C. This allowed separation of the GST fragment of the fusion proteins, which remained bound to the gel matrix from the metal-HpMT portions that were eluted together with thrombin. Therefore, the eluate was concentrated using Centriprep Concentrators (Amicon; Millipore, MA, USA) with a cut-off of 3 kDa and subsequently fractionated using fast protein liquid chromatography (FPLC), through a Superdex-75 column (GE Healthcare) equilibrated with 50 mM Tris-HCl, pH 7.0, and run at 1 mL min^-1^. Fractions were collected and analysed for protein content by their absorbance at 254 nm. Aliquots of the protein-containing FPLC fractions were analysed by 15% SDS-PAGE and stained by Coomassie Blue. HpMT-containing samples were pooled and stored at -70°C until further use. Due to the specific recombinant expression conditions, the three synthesized snail MT isoforms contained one additional amino acid residue (G) at their N-termini in relation to the native isoforms previously isolated [20]. These modifications do not interfere with the metal-binding capacity, as previously shown for both vertebrate [61] and invertebrate [8] MT isoforms.

### Analysis of recombinantly expressed and *in vitro *prepared metal-HpMT complexes

The recombinantly expressed metal-MT complexes were analysed for element composition (S, Zn, Cd and Cu) by inductively coupled plasma atomic emission spectroscopy (ICP-AES) on a Polyscan 61E spectrometer (Thermo Jarrell Ash Corporation, MA, USA) at appropriate wavelengths (S, 182.040 nm; Zn, 213.856 nm; Cd, 228.802 nm; Cu, 324.803 nm), either under 'conventional' (dilution with 2% HNO_3 _(v/v)) or under 'acidic' (incubation in 1 M HCl at 65°C for 5 min) conditions [62]. MT concentration in the recombinant preparations was calculated from the acidic ICP sulphur measurements, thus assuming the only contribution to their S content was that made by the HpCuMT and HpCdMT peptides. Protein concentrations were confirmed by standard amino acid analysis performed on an Alpha Plus Amino acid Autoanalyzer (Pharmacia LKB Biotechnology, Uppsala, Sweden) after sample hydrolysis in 6 M HCl (22 h at 110°C). Ser, Lys and Gly contents were used to extrapolate sample concentrations.

CD spectroscopy was performed using a model J-715 spectropolarimeter (JASCO, Gross-Umstadt, Germany) equipped with a computer (J-700 software, JASCO). Measurements were carried out at a constant temperature of 25°C maintained by a Peltier PTC-351 S apparatus (TE Technology Inc, MI, USA). Electronic absorption was measured on an HP-8453 diode-array ultra violet (UV)-vis spectrophotometer (GMI Inc, MN, USA), using 1-cm capped quartz cuvettes, and correcting for the dilution effects by means of the GRAMS 32 software (Thermo Fisher Scientific Inc, MA, USA).

Molecular mass determination was performed by electrospray ionization mass spectrometry equipped with a time-of-flight analyser (ESI-TOF MS) using a Micro Tof-Q Instrument (Brucker Daltonics GmbH, Bremen, Germany) calibrated with NaI (200 ppm NaI in a 1:1 H_2_O: isopropanol mixture), interfaced with a Series 1100 HPLC pump (Agilent Technologies, CA, USA) equipped with an autosampler, both controlled by the Compass Software. The experimental conditions for analysing proteins with divalent metals (Zn, Cd) were: 20 μL of the sample were injected through a PEEK long tube (1.5 m × 0.18 mm i.d.) at 40 μL/min under the following conditions: capillary-counterelectrode voltage, 5.0 kV; desolvation temperature, 90-110°C; dry gas 6 L/min. Spectra were collected throughout an m/z range from 800 to 2000. The proteins that contain copper were analysed injecting 20 μL of the sample at 30 μL/min; capillary-counterelectrode voltage, 4.0 kV; desolvation temperature, 80°C; m/z range from 800 to 2000. The liquid carrier was a 90:10 mixture of 15 mM ammonium acetate and acetonitrile, pH 7.0. For the analysis at acidic pH the conditions used were the same as those used in the analysis of the case for divalent metals, except in the composition of the carrier liquid which, in this case, was a 95:5 mixture of formic acid and acetonitrile at pH 2.4. All samples were injected at least in duplicate to ensure reproducibility. In all cases, molecular masses were calculated according to the reported method [63].

Metal replacement titrations were performed by adding the corresponding metal ions (Cd^2+ ^or Cu^+^) at equivalent molar ratios to the recombinant Zn-HpMT complexes. Titrations were carried out following previously described procedures [64,65]. The resulting *in vitro *complexes were analysed by UV-Vis and CD spectroscopy as well as mass spectrometry. All assays were carried out in an Ar atmosphere and the pH for all experiments remained constant throughout, without the addition of any extra buffers.

### Metal tolerance complementation assays in transformed yeast MT-knockout cells

The *Saccharomyces cerevisiae *DTY4 strain (*MATα, leu2-3, 112his3*^*Δ*^*1, trp1-1, ura3-50, gal1, cup1::URA3*) was used for metal tolerance complementation assays. This strain is characterized by a total MT deficiency due to *cup1 *disruption and *Crs5 *truncation [66].

The cDNAs coding for the different MTs assayed - the two snail MT isoforms (snail HpCdMT and snail HpCuMT), the two yeast MTs (Cup1 and Crs5) and the mouse MT1 - were ligated into the *Bam*HI/*Pst*I sites of the yeast vector p424, which contains TRP1 for selection, the constitutive GPD (glyceraldehyde-3-phosphate dehydrogenase) promoter for heterologous gene expression, and the CYC1 (cytochrome-c-oxidase) transcriptional terminator [67]. The recombinant p424 vectors were introduced into the DTY4 cells using the lithium acetate procedure [68]. Transformed cells were selected according to their capacity to grow in synthetic complete medium (SC) without Trp and Ura.

For metal tolerance tests, transformed yeast cells were initially grown in selective SC-Trp-Ura medium at 30°C and 220 rpm until saturation. These cells were then diluted to OD_600 _0.01 and used to re-inoculate tubes with 3 mL of fresh medium supplemented with CuSO_4 _added at 15, 30, 45, 60, 75, 90 and 105 μM final concentrations or CdCl_2 _at 1, 2, 4, 6 and 8 μM final concentrations. These cultures were allowed to grow for 18 h and the final OD_600 _was recorded and plotted as a percentage of the OD_600 _reached by the culture grown without metal supplement. For each concentration, and each kind of transformation, two replicas were run.

### MT sequence alignment and phylogenetic analyses

MT amino acid sequences used for pulmonate MT alignments were derived mostly from amino acid sequence files or translated cDNA open reading frame sequences published in GenBank (http://www.ncbi.nlm.nih.gov/Tools/; see accession numbers in the legend to Figure 9). The editing and alignment were done manually in combination with ClustalX software Version 2.0.9 [69]. For phylogenetic analyses, nucleotide sequences of the coding region of mollusc MT cDNAs or MT genes as well as protein primary sequences were used as published in GenBank (for accession numbers see legend of Figure 9). Phylogenetic reconstructions we performed with neighbour-joining [70] using the computer program PAUP* (version 4). The robustness of the phylogenetic hypothesis was tested by bootstrapping (1000 replicates) [71].

## Abbreviations

ATG: anti-thymocyte globulin; CD: circular dichroism; cDNA: complimentary DNA; ESI: electrospray ionization; FPLC: fast protein liquid chromatography; GPD: glyceraldehyde-3-phosphate dehydrogenase; GST: glutathione-S-transferase; Hp: *Helix pomatia*; ICP-AES: inductively coupled plasma atomic emission spectroscopy; ISH: *in situ *hybridization; GPD: glyceraldehyde-3-phosphate dehydrogenase; mRNA: messenger RNA; MS: mass spectrometry; MT: metallothionein; PBS: phosphate buffered saline; RT-PCR: real time polymerase chain reaction; SC: synthetic complete medium.

## Authors' contributions

ÒP, AP, and SPR carried out the recombinant expression studies, participating in the construction of the expression vectors, the synthesis and characterization of the corresponding metal complexes, and also performed the yeast complementation experiments. ME and MH performed quantitative Real-Time PCR experiments and in situ hybridization, and carried out most of the sequencing work of MT cDNAs from different pulmonate snail species. AB calculated and established the phylogenetic trees. MC and SA designed the study together with RD, supervised recombinant DNA and recombinant protein experiments, yeast complementation studies, as well as analytical, spectrometric and spectrophotometric work, and contributed to the drafting of the manuscript. RD supervised and participated in molecular sequencing and Real-Time PCR as well as in situ hybridization, and performed chromatography and Reversed-Phase HPLC. He was responsible for the protein alignments and assisted in calculation of phylogenetic trees. He designed the study together with MC and SA, and drafted the manuscript. All authors read and approved the final manuscript.
